# Disease- and headache-specific microRNA signatures and their predicted mRNA targets in peripheral blood mononuclear cells in migraineurs: role of inflammatory signalling and oxidative stress

**DOI:** 10.1186/s10194-022-01478-w

**Published:** 2022-09-02

**Authors:** Timea Aczél, Bettina Benczik, Bence Ágg, Tamás Körtési, Péter Urbán, Witold Bauer, Attila Gyenesei, Bernadett Tuka, János Tajti, Péter Ferdinandy, László Vécsei, Kata Bölcskei, József Kun, Zsuzsanna Helyes

**Affiliations:** 1 Department of Pharmacology and Pharmacotherapy, Medical School & Szentágothai Research Centre, Molecular Pharmacology Research Group, Centre for Neuroscience, University of Pécs, Pécs, Hungary; 2 Cardiometabolic and MTA-SE System Pharmacology Research Group, Department of Pharmacology and Pharmacotherapy, Semmelweis University, Budapest, Hungary; 3 Pharmahungary Group, Szeged, Hungary; 4 MTA-SZTE Neuroscience Research Group, University of Szeged, Szeged, Hungary; 5 Faculty of Health Sciences and Social Studies, University of Szeged, Szeged, Hungary; 6 Szentágothai Research Centre, Bioinformatics Research Group, Genomics and Bioinformatics Core Facility, University of Pécs, Pécs, Hungary; 7 Department of Neurology, Faculty of Medicine, Albert Szent-Györgyi Clinical Center, University of Szeged, Szeged, Hungary; 8 PharmInVivo Ltd., Pécs, Hungary; 9 Department of Pharmacology and Pharmacotherapy, University of Pécs Medical School, Szigeti út 12, 7624 Pécs, Hungary

**Keywords:** Migraine, miRNA, Peripheral blood mononuclear cells, Cytokines, Oxidative stress, Human

## Abstract

**Background:**

Migraine is a primary headache with genetic susceptibility, but the pathophysiological mechanisms are poorly understood, and it remains an unmet medical need. Earlier we demonstrated significant differences in the transcriptome of migraineurs' PBMCs (peripheral blood mononuclear cells), suggesting the role of neuroinflammation and mitochondrial dysfunctions. Post-transcriptional gene expression is regulated by miRNA (microRNA), a group of short non-coding RNAs that are emerging biomarkers, drug targets, or drugs. MiRNAs are emerging biomarkers and therapeutics; however, little is known about the miRNA transcriptome in migraine, and a systematic comparative analysis has not been performed so far in migraine patients.

**Methods:**

We determined miRNA expression of migraineurs’ PBMC during (ictal) and between (interictal) headaches compared to age- and sex-matched healthy volunteers. Small RNA sequencing was performed from the PBMC, and mRNA targets of miRNAs were predicted using a network theoretical approach by miRNAtarget.com™. Predicted miRNA targets were investigated by Gene Ontology enrichment analysis and validated by comparing network metrics to differentially expressed mRNA data.

**Results:**

In the interictal PBMC samples 31 miRNAs were differentially expressed (DE) in comparison to healthy controls, including hsa-miR-5189-3p, hsa-miR-96-5p, hsa-miR-3613-5p, hsa-miR-99a-3p, hsa-miR-542-3p. During headache attacks, the top DE miRNAs as compared to the self-control samples in the interictal phase were hsa-miR-3202, hsa-miR-7855-5p, hsa-miR-6770-3p, hsa-miR-1538, and hsa-miR-409-5p. MiRNA-mRNA target prediction and pathway analysis indicated several mRNAs related to immune and inflammatory responses (toll-like receptor and cytokine receptor signalling), neuroinflammation and oxidative stress, also confirmed by mRNA transcriptomics.

**Conclusions:**

We provide here the first evidence for disease- and headache-specific miRNA signatures in the PBMC of migraineurs, which might help to identify novel targets for both prophylaxis and attack therapy.

**Supplementary Information:**

The online version contains supplementary material available at 10.1186/s10194-022-01478-w.

## Introduction

Migraine is a common primary headache disease with complex pathophysiological mechanisms. This neurovascular disorder involves meningeal vasodilatation, oedema formation activation and sensitisation of the trigeminal pain pathways [1]. Immune activation and the release of proinflammatory cytokines and neuropeptides, such as CGRP (calcitonin gene-related peptide) and PACAP (pituitary adenylate cyclase-activating polypeptide), seem to be critical elements [2–6]. Besides genetic factors, environmental influence has been described for migraine susceptibility [7]. There has not been a breakthrough in effective personalised preventive or acute treatment of migraine despite extensive efforts. It is still an unmet medical need mainly due to the heterogeneity of the disease. Therefore, it is essential to explore the complexity of the pathophysiological mechanisms by unbiased omics approaches. PBMCs, containing lymphocytes (T cells, B cells, natural killer cells) and monocytes, have become attractive blood-based marker candidates in clinical practice due to minimally invasive sampling and relatively simple isolation. Their potential value consists of reflecting pathophysiological changes in the central nervous system in various diseases. Hence neuroinflammatory processes might be studied in a specific way using PBMCs [8–10]. We have recently provided evidence for increased immune cell activity, oxidative stress and mitochondrial dysfunction in migraine using transcriptomics of peripheral blood mononuclear cells (PBMC) [11]. MicroRNAs (miRNAs) are short, non-coding RNAs regulating post-transcriptional gene expression, and thus controlling cell-to-cell communication and multiple cellular processes. [12–14]. Since miRNAs can reflect the environmental impact on gene expression modulation [15], their relevance in preclinical research and clinical applications is rapidly growing. Circulating miRNAs are stable, easily measurable and tissue-specific molecules [13, 16]; therefore, they receive increased attention as tissue-specific biomarkers for various pathological processes, drug resistance modulators, and novel drug targets. Several clinical studies suggest specific miRNAs to be indicators of disease progression, e.g. in diabetes, coronary heart disease, breast cancer, epilepsy, depressive disorder, stroke, etc. [16]. Interestingly, miRNAs can interfere with immune, vascular, and neuronal activities [17–19]. Thus, they might be valuable in neuro-immune-vascular interactions and alterations like neuropathic pain, Alzheimer’s and Parkinson’s disease, multiple sclerosis and migraine [20]. Their relevance in chronic pain conditions, e.g. complex regional pain syndrome, irritable bowel syndrome and fibromyalgia, have also been studied [21, 22].

Detection of miRNAs in molecular research and diagnostics is fundamental as well as challenging due to their low cell content, small size, and the similar sequences of miRNA families. Continuous development has yielded detection methods ranging from conventional techniques such as microarrays and PCR (polymerase chain reaction) to the more recent next-generation sequencing. MicroRNA microarray is a technique for miRNA profiling utilizing hybridization between target miRNAs and their corresponding complementary probes on a solid surface and eventual detection of the signal intensity of the hybridization probes. It has high throughput although low sensitivity and selectivity. PCR, on the other hand, has high sensitivity, a wider range but poor specificity for miRNAs, along with limited throughput. RNA-seq represents the last extensive approach for miRNA profiling. Its workflow involves the isolation of RNA followed by cDNA (complementary DNA) library construction and sequencing. Small RNA-Seq was chosen for our study based on its main advantages of having a non-targeted and greatly sensitive nature [23, 24].

The potential roles of miRNAs in migraine diagnosis and therapy have recently been reviewed, but the clinical relevance of the results needs to be clarified and confirmed [25, 26]. Few investigations have been performed on specific miRNAs [16–19] and microarray-based miRNA profile descriptions, with controversial results [27–29]. One study describes serum miRNA alterations both during attacks and pain-free periods of migraineurs, using high-content serum microRNA PCR arrays [27]. In contrast, no alterations were described in the miRNA profiles of 20 mixed gender migraine patients’ PBMC samples compared to 5 headache-free controls in a conference abstract [29]. The extension of this pilot study (MicroMIG) demonstrated a few DE miRNAs when comparing different subgroups of migraine patients to controls, but they were not specified [30]. Two-sample microarray analysis of exosomes isolated from pooled blood samples of 15 female migraineurs without aura compared to 13 healthy matched controls described 22 dysregulated circulating miRNAs, 4 of which were validated by qPCR. MiR-22 and let-7b downregulation was confirmed in PBMC samples [28]. PBMCs represent an easily accessible biological material, which can reflect pathophysiological changes in the brain and characterize neuroinflammatory processes [8, 9]. Therefore, here we determine miRNAs from PBMC samples to gather more information on disease- and headache-related mechanisms in migraineurs since their transcriptomic alterations were proven to be specific and sensitive indicators of migraine-related processes [11]. To our knowledge, this is the first study to perform small RNA sequencing on PBMCs isolated from interictal and ictal samples of migraine patients compared with healthy controls. Combining network theoretical miRNA-target prediction and an already analyzed mRNA data set provides a unique and valuable platform for discovering potential novel biomarkers and/or drug targets in migraine.

## Methods

### Study design

The study was approved by the National Public Health Center, Ministry of Human Capacities (28324–5/2019/EÜIG). Informed written consent was obtained from each participant following the Declaration of Helsinki.

Sixteen episodic migraine patients with (*n* = 1) or without aura (*n* = 15) and 12 healthy controls, aged between 23 and 59 years, were enrolled in the present study. Out of the 16 migraineurs, 8 patients applied for blood sampling during headache attacks (one patient applied twice). Therefore, 9 ictal (self-controlled paired) samples were further investigated. In total, 37 samples from 28 participants were selected from the cohort collected between September 2018 and December 2019. The inclusion criteria were female gender, aged 20–60 years, diagnosed with migraine with or without aura by the third edition of the International Classification of Headache Disorders [31]. Exclusion criteria were chronic inflammatory diseases and depression. A detailed questionnaire was used to assess the characteristics of the headache concerning the following: prophylactic or attack medication before sampling, the number of attacks in the previous month, the time of the last episode, the beginning of the current attack, other known diseases, applied drugs and contraceptives, the relation of migraine attacks to the menstrual cycle, the presence of allodynia, attack frequency, duration of migraine, severity of pain during attacks as measured on a visual analogue scale, co-morbidities with other chronic diseases, the familial manifestation of migraine, regular sport activity and the time of the last meal were recorded, as described earlier [11]. Besides, patients were examined by Hamilton’s depression scale to exclude participants with an assessment of depression. There were no restrictions regarding food and drink intake. Blood samples were collected from migraine sufferers in an attack-free period and during an attack. Healthy controls were screened for non-reported/non-treated headaches and matched all demographic characteristics.

### Sample collection

Human blood samples were collected from participants, after which processing started within 1 h. Tubes containing anticoagulant EDTA (ethylenediaminetetraacetic acid) were used for sample collection. PBMCs were isolated using Ficoll-Paque PREMIUM (GE Healthcare, Budapest, Hungary) according to the manufacturer's instructions, as previously described [11]. After removing the liquid phase, cells were resuspended with 1 ml of TRI Reagent (Molecular Research Center, Cincinnati, OH, USA) and stored at -80 °C until further investigations.

### RNA extraction and quality control

Total RNA was extracted by applying the phenol–chloroform-based TRI Reagent procedure (Molecular Research Center, Cincinnati, OH, USA), followed by extraction and purification using the Direct-zol RNA MiniPrep kit (Zymo Research, Irvine, CA, USA). The manufacturer’s protocol was followed, including the optional on-column DNase digestion, as described earlier [11, 32]. RNA concentrations were measured with Qubit 3.0 (Invitrogen, Carlsbad, CA, USA), and quality was checked on TapeStation 4200 using RNA ScreenTape (Agilent Technologies, Santa Clara, CA, USA). High-quality (RIN > 8) RNA samples were further used for library preparation.

### Small RNA library preparation and sequencing

Small RNA Libraries were generated using the SmallRNA-Seq Library Prep Kit (Lexogen, Vienna, Austria). Briefly, 200 ng of total RNA was used as input, and 3’ and 5’ adapters were ligated, followed by column purifications. Thereafter, the ligation products were reverse transcribed, and the input RNA, flanked by 5’ and 3’ adapters, were converted into cDNA. Finally, the libraries were amplified and barcoded using 16 cycles of PCR. All libraries were assessed on the TapeStation 4200 (Agilent Technologies, Santa Clara, CA, USA) to examine if adapter dimers formed during PCR. The libraries were sequenced on an Illumina NextSeq550 platform generating 75 bp single-end reads. All RNA-Seq datasets generated as part of this study will be publicly available at the European Nucleotide Archive (https://www.ebi.ac.uk/ena), under accession number PRJEB46142. A total of 37 blood samples were used for PBMC small RNA sequencing.

### Bioinformatics – miRNA-Seq, miRNA target prediction, and miRNA-mRNA target network analysis

The sequencing reads were aligned against the *Homo sapiens* reference genome (GRCh37 Ensembl release) with STAR v2.5.3a [33]. After alignment, the reads were associated with known protein-coding genes, and the number of reads aligned within each gene was counted using Rsubread package v2.0.0 [34]. Gene count data were normalized using the TMM (trimmed mean of M values) normalization method of the edgeR R/Bioconductor package (v3.28, R v3.6.0, Bioconductor v3.9) [35]. The data were further log transformed using the voom approach [36] in the limma package [37] for statistical testing. Normalized counts were represented as TPM (transcripts per million) values. FC (fold change) values between the compared groups resulting from the linear modelling process and modified t-test p-values were produced by the limma package. The Benjamini–Hochberg method was used to control the FDR (False Discovery Rate), and adjusted p-values were calculated by limma. In the case of paired ictal and interictal samples, the correlation between samples originating from the same patient was taken into account using the *duplicateCorrelation* function of limma.

After statistical filtering for DE (differentially expressed) miRNAs in each comparison (|FC|> 1.2 and *p* < 0.05), the DIANA-miRPath v3.0 web tool [38] was used to perform GO (Gene Ontology) and KEGG (Kyoto Encyclopedia of Genes and Genomes) enrichment analysis (*p* < 0.01) using the microTCDS predicted targets of the DE miRNA list for each comparison with default settings, accessed on November 12th, 2021. BP (biological process) ontology GO results were further filtered for redundant terms using Revigo [39], with default settings accessed on November 14th, 2021. To highlight specific terms, GO BP terms with frequency values less than 0.15 were taken into account, where the frequency is defined as the proportion of gene products annotated by the selected GO term in the European Bioinformatics Institute Gene Ontology Annotation database for human. Higher frequency value implies more general terms, and lower implies more specific ones.

The network theoretical miRNAtarget.com™ software (mirnatarget.com; Pharmahungary, Szeged, Hungary) was used to predict target genes and their expected expression changes based on the lists of DE miRNAs. For this analysis, similarly to previous works [18, 19, 40–43], data from the manually curated, experimentally validated miRTarBase [44] (v7.0), the predicted miRDB [45] (v5.0 with score > 80.0) and microRNA.org [46] (release of August 2010 with mirSVR score < -1.2), miRNA-target interaction databases were integrated by miRNAtarget. Positive and negative edge weights (1, -1) in the network, referring to predicted interaction with up- and downregulated miRNAs, respectively, were summed to calculate node strength values, which estimate the extent of the predicted expression change of the targets. To achieve adequate visual representation of the information content of our networks, we used the EntOptLayout plugin [47] (version 2.1) for the Cytoscape [48] (version 3.7.2) software and further improved the visualisation by graphically highlighting node strength values. To elucidate the role of predicted target genes in biological processes, GO enrichment analysis [49, 50] was performed for each comparison, separately for up-and downregulated targets. The online PANTHER Overrepresentation Test ([51], version released on July 28^th^, 2020) was used with default settings (Fisher’s Exact test with FDR calculation). From the GO Ontology Database ([52], version released on October 9^th^, 2020) *Homo sapiens* “GO biological process complete” annotation dataset was used for the analysis.

### Validation of predictions at mRNA level based on RNA sequencing data

To validate the small RNA sequencing-based miRNA-target predictions at the mRNA level, we used mRNA-Seq data from our previous study [11]. We included samples from the same cohort mentioned above in the present investigation with minimal changes to ensure a more homogenous patient group. We validated the in silico predicted miRNA-target interactions at the mRNA level by matching the predicted mRNAs from the present study to the differentially expressed mRNAs from our previous study cited above. We compared the predicted node strength values to the mRNA binary logarithm of the FC values with respect to the direction of changes. We considered genes to be validated that showed expression change consistent with the predictions.

### Statistical analysis

The demographic and clinical characteristics of the studied population are presented in Table 1. Numerical data were summarized with their arithmetic means and 95% confidence intervals. Continuous parameters were tested for normality with Shapiro–Wilk’s tests and visual inspections. Homogeneity of variance across groups was studied using Levene's tests. If the resulting p-values from these two tests were less than the significance level of 0.05, non-parametric tests were used for response variables. The differences between the mean values of each parameter and its distribution within the study population (migraineurs and healthy) were tested using the non-parametric Kruskal–Wallis rank-sum test or ANOVA (analysis of variance), if applicable followed by Benjamini–Hochberg multiple-testing correction. To compare the parameters between the migraineurs group and its two phases: interictal and ictal, paired-samples Wilcoxon test (or paired t-test, if applicable) was used. The statistical significance level was set at 0.05 for all two-sided tests. The data were analysed using R version 4.0.2.

**Table 1:** Description of the study subjects, main demographic, and clinical characteristics. Mean values (with 95% confidence intervals) of the selected parameters in migraineurs (interictal and ictal phase) and healthy control groups. Headache pain was evaluated with the VAS (visual analogue scale). Categories are as follows: 1–4 grade (1), 5–7 grade (2), 8–10 grade (3)

	**Interictal** **(*n* = 16)**	**Ictal** **(*n* = 8)**	**Healhy control** **(*n* = 1**2**)**	***P*—value**
**Age**	34.62 (28.66—40.58)	36.87 (26.93—46.81)	30.75 (27.95—33.55)	0.91
**Body mass index (BMI)**	21.95 (19.84—24.06)	21.90 (19.59—24.20)	23.28 (21.39—25.17)	0.36
**Last meal (hours ago)**	6.67 (3.65—9.68)	6.62 (1.64—11.61)	3.83 (0.66—6.99)	0.21
	**Co-morbidities and therapy**			
**Known other diseases (%)**	50 (24—75)	50 (12—87)		1.00
**Regular medication (except attack therapy) (%)**	31 (7—54)	38 (1—73)		0.76
**Hormonal contraceptives (%)**	37 (12—62)	25 (0—57)	25 (0—51)	0.73
**Antimigraine prophylactic therapy (%)**	0	0		
	**Clinical features of the headache**			
**Disease duration (years)**	13.18 (8.95—17.41)	15.5 (7.98—23.01)		0.51
**Attack frequency (attack/year)**	23.93 (16.65—31.22)	21.87 (10.95—32.79)		0.62
**Visual analogue scale category (VAS)**	2.68 (2.45—2.92)	2.62 (2.26—2.98)		0.76
**Allodynia (%)**	31 (7—54)	50 (12—87)		0.38
**Chronic pain (%)**	18 (0—38)	12 (0—37)		0.70
**Menstruation-headache relationship (sensitive) (%)**	37 (12—62)	37 (1—73)		1.00
**Migraineurs in the family (%)**	68 (0.45—0.92)	62 (26—98)		0.76
**Regular exercise (%)**	56 (30—81)	62 (26—98)	17 (0—39)	0.06
	**Features of attacks before samplings**			
**Number of attacks in the previous month**	2.62 (1.4—3.84)	0.87 (0.18—1.56)		0.03
**Last attack (days ago)**	20.25 (3.26—37.23)	36.62 (3.38—69.86)		0.04
**Beginning of attack before ictal sampling (hours)**		6.31 (1.24—11.38)		
**Actual VAS**		5.62 (4.79—6.45)		

## Results

### Clinical characteristics of the patient population

The patients’ demographic and main clinical features for the total number of 28 participants are provided in Table 1. The interictal group consists of 16 migraine patients, with blood sampling in a headache-free period. Ictal samples were collected from 8 of them for self-control comparison. The 12 control samples consisted of healthy volunteers. Demographic characteristics were similar across the subgroups. No significant alterations were found when comparing different subgroups' demographic and clinical characteristics, e.g. age, attack frequency, pain severity, etc. The only significant difference between the interictal and ictal groups was the number of attacks in the previous month and the date of the last attack before sampling (Table 1).

### microRNA signatures of PBMC samples

When interictal PBMC samples were compared to healthy ones, 31 miRNAs were DE, when significance was tested against a fold change threshold of 1.2 and a p-value threshold of 0.05. 14 miRNAs were upregulated, and 17 were downregulated. Based on the average of fold change and p-value ranks (average rank), hsa-miR-5189-3p, hsa-miR-96-5p, hsa-miR-3613-5p, hsa-miR-99a-3p, and hsa-miR-542-3p were found at the top of the DE miRNA list (Table 2; for further details please see Table S1).

In ictal vs interictal comparison, 25 miRNAs were DE (significance was tested against fold change 1.2 and p-value: 0.05), 15 were upregulated, and 10 were downregulated. Top 5 miRNAs were as follows: hsa-miR-3202, hsa-miR-7855-5p, hsa-miR-6770-3p, hsa-miR-1538, hsa-miR-409-5p (Table 3; for further details please see Table S2).

**Table 2:** DE miRNAs in interictal PBMC samples compared to healthy ones. Differential expression was tested against a fold change threshold of 1.2 and a *p*-value threshold of 0.05. Average rank was calculated as the mean ranks of miRNAs based on fold change and p-value

ID	Fold change	*P*-value	Average rank
hsa-miR-5189-3p	2,59	0,0057	1
hsa-miR-96-5p	-2,40	0,0032	2
hsa-miR-3613-5p	2,55	0,0101	3
hsa-miR-99a-3p	2,37	0,0079	4
hsa-miR-542-3p	2,40	0,0164	5
hsa-miR-6803-3p	2,19	0,0162	6
hsa-miR-6731-3p	-2,14	0,0084	7
hsa-miR-577	-2,17	0,0200	8
hsa-miR-95-3p	-2,06	0,0184	9
hsa-miR-556-3p	-2,18	0,0228	10
hsa-miR-412-5p	-2,36	0,0290	11
hsa-miR-5701	-2,24	0,0263	12
hsa-miR-3064-5p	2,10	0,0247	13
hsa-miR-196a-5p	-2,55	0,0450	14
hsa-miR-5189-5p	1,93	0,0222	15
hsa-let-7i-3p	-1,82	0,0067	16
hsa-miR-1277-5p	2,07	0,0402	17
hsa-miR-29b-3p	-1,85	0,0214	18
hsa-miR-4676-3p	1,87	0,0261	19
hsa-miR-548j-3p	1,91	0,0453	20
hsa-miR-1260b	1,78	0,0361	24
hsa-miR-326	1,62	0,0027	26
hsa-miR-3174	1,79	0,0480	27
hsa-miR-210-3p	1,77	0,0461	31
hsa-miR-32-5p	-1,65	0,0373	34
hsa-miR-342-3p	-1,60	0,0307	40
hsa-miR-3607-3p	-1,59	0,0381	44
hsa-miR-142-5p	-1,54	0,0239	54
hsa-miR-192-5p	-1,56	0,0440	57
hsa-miR-155-5p	-1,43	0,0186	74
hsa-let-7 g-5p	-1,43	0,0351	76

In ictal samples compared to the healthy group, 31 miRNAs were DE (fold change: 1.2, *p*-value: 0.05), 22 were upregulated, 9 were downregulated, hsa-miR-1277-5p being at the top of the list (Table S3).

**Fig. 1:**
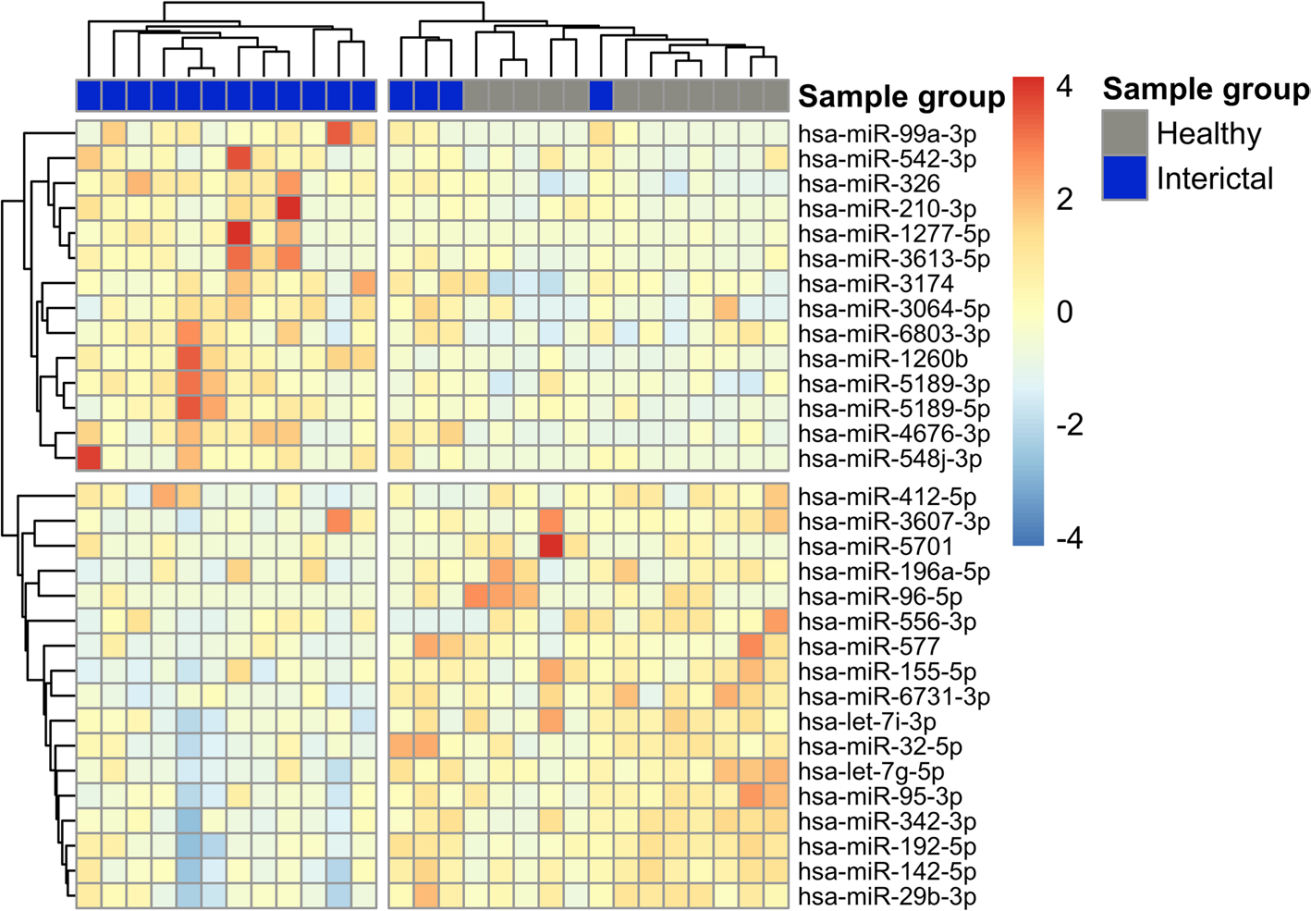
Heat map representation of differentially expressed genes in the interictal vs healthy PBMC comparison. Columns represent samples, and rows represent genes. Pearson correlation was respectively calculated between samples and genes, visualised by dendrograms

**Fig. 2:**
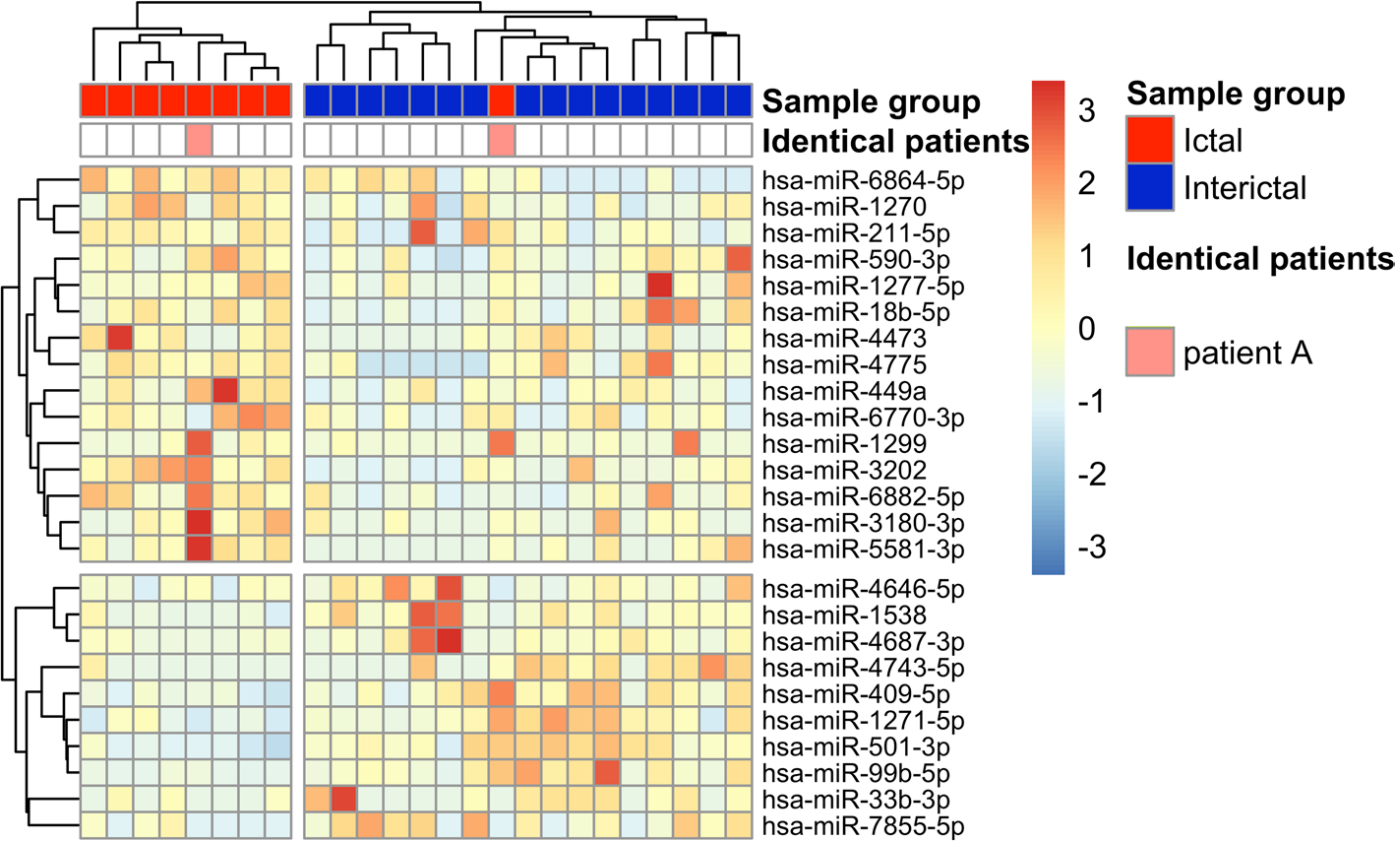
Heat map representation of differentially expressed genes in the ictal vs interictal comparison. Columns represent samples, and rows represent genes. Pearson correlation was respectively calculated between samples and genes, visualised by dendrograms. Samples from patient “A” in different ictal and interictal periods are marked with respective colors

DE miRNAs were visualised on heat maps (Figs. 1 and 2), and samples were clustered based on correlation. They were partitioned into major groups, largely overlapping with the original patient groups. DE miRNAs for ictal vs. healthy interaction were represented on a heat map in Figure S1.

Functional enrichment analysis of DE miRNAs (DE list enrichment) was carried out. GO, and KEGG analysis was performed to link information with functions and utilities of the biological system (Tables 4 and 5). Interictal samples compared to healthy ones show a relation to several GO categories (pathways and processes), including several toll-like receptor signalling pathways, platelet degranulation, cellular response to glucagon stimulus, and alpha-linolenic acid metabolic process.

**Table 3:** DE miRNAs in ictal PBMC samples compared to interictal ones. Differential expression was tested against a fold change threshold of 1.2 and a p-value threshold of 0.05. Average rank was calculated as the mean ranks of miRNAs based on fold change and p-value

ID	Fold change	*P*-value	Average rank
hsa-miR-3202	2,94	0,0014	1
hsa-miR-7855-5p	-2,69	0,0033	2
hsa-miR-6770-3p	2,90	0,0135	3
hsa-miR-1538	-2,22	0,0023	4
hsa-miR-409-5p	-2,57	0,0072	5
hsa-miR-501-3p	-2,16	0,0014	6
hsa-miR-1299	3,53	0,0248	7
hsa-miR-1271-5p	-2,60	0,0187	8
hsa-miR-4687-3p	-2,21	0,0217	9
hsa-miR-4743-5p	-2,07	0,0135	10
hsa-miR-1277-5p	2,08	0,0184	11
hsa-miR-3180-3p	2,51	0,0351	12
hsa-miR-4646-5p	-2,18	0,0311	13
hsa-miR-5581-3p	2,14	0,0251	14
hsa-miR-6882-5p	2,11	0,0257	15
hsa-miR-449a	1,94	0,0332	16
hsa-miR-4473	2,01	0,0439	17
hsa-miR-4775	1,96	0,0422	18
hsa-miR-33b-3p	-1,92	0,0411	20
hsa-miR-99b-5p	-1,84	0,0242	21
hsa-miR-1270	1,87	0,0328	22
hsa-miR-18b-5p	1,94	0,0463	23
hsa-miR-6864-5p	1,90	0,0374	24
hsa-miR-211-5p	1,91	0,0493	25
hsa-miR-590-3p	1,82	0,0392	31

In the ictal PBMC samples compared to the interictal group, several toll-like receptor signalling pathways and positive regulation of protein insertion into the mitochondrial membrane involved in the apoptotic signalling pathway were altered. Interestingly, regulation of rhodopsin mediated signalling pathway and phototransduction were significantly affected.

KEGG pathway mapping revealed ECM (extracellular matrix)—receptor interaction and GABAergic synapse involvement, characterising interictal samples compared to control samples. TGF-beta) Transforming growth factor β and ErbB (epidermal growth factor receptor) signalling pathways appeared in interictal vs healthy and ictal vs interictal comparisons.

### Predicted targets with the highest absolute node strength values in interictal vs healthy comparison

**Fig. 3:**
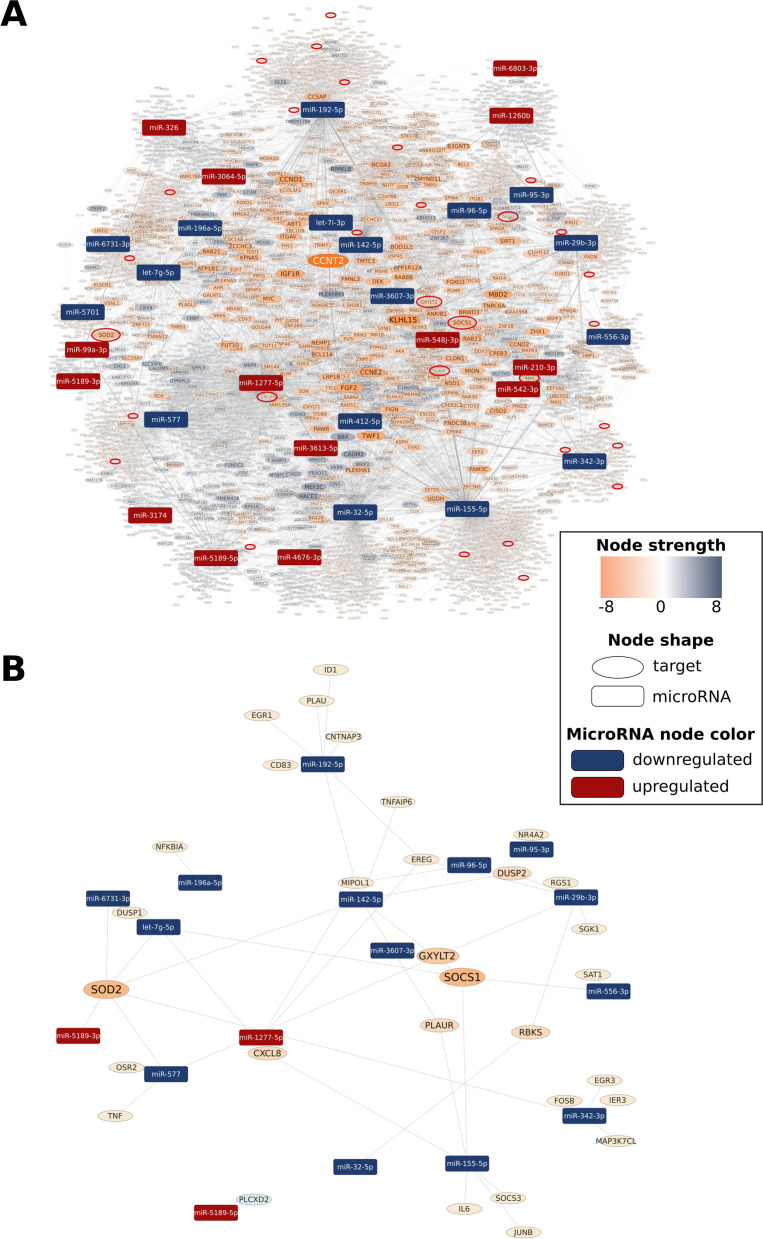
Visualisation of miRNA-mRNA interaction network (EntOptLayout) and target prediction analysis by miRNAtarget.com™ (interictal vs healthy). Rectangle and oval-shaped nodes represent miRNAs and mRNA targets of miRNAs, respectively. The node size and colour intensity of mRNA targets change according to node strength values. Down (blue)- and upregulated (red) interacting miRNAs suggest a central role of upregulated (orange) cyclin T2 (CCNT2) and kelch like family member 15 (KLHL15) and downregulated (light-blue) cell adhesion molecule 2 (CADM2) mRNAs. Whole predicted miRNA-target interaction network is shown on panel **A**. To highlight important mRNA targets, targets with an absolute node strength value less than or equal to 1 (i.e. -1, 0 or 1) presented uniformly smaller and fainter. On panel **B***,* a subnetwork of the whole predicted miRNA-target interaction network is shown, containing only those target mRNAs (marked with a red oval on panel **A**) and their interacting miRNAs that were validated by RNA sequencing. The same arrangement as in the whole network with a proportional magnification of the target mRNAs was applied

7012 mRNA targets of the 31 DE miRNAs were predicted when comparing interictal with healthy control samples. Predicted targets with the highest absolute node strength values are enlisted in Table 6. and the intersect of predicted targets and DE mRNAs in S4. As compared to the control group, targets like CADM2 (cell adhesion molecule 2), PLEKHM3 (pleckstrin homology domain-containing M3), MEF2C (myocyte enhancer factor 2C), BBX (BBX high mobility group box domain containing), ribosomal modification protein RIMKLB (rimK like family member B) and HACE1 (HECT domain and ankyrin repeat containing E3 ubiquitin protein ligase 1) were significantly downregulated. In contrast, CCN T2 (cyclin T2) and KLHL15 (kelch like family member 15) showed significant upregulation in the interictal group (Table 5). DE miRNAs with their predicted interactions are shown in Fig. 3.

**Table 4:** Top 15 pathways containing the list of KEGG results from the Mirpath v3 webtool analysis

**Interictal vs healthy**				**Ictal vs interictal**			
**KEGG pathway**	***p*-value**	**Number of genes**	**Number of miRNAs**	**KEGG pathway**	***p*-value**	**Number of genes**	**Number of miRNAs**
ECM-receptor interaction	6.11E-10	47	20	Proteoglycans in cancer	4.7E-07	122	18
Proteoglycans in cancer	2.60E-08	114	23	Prion diseases	5.1E-05	16	11
PI3K-Akt signalling pathway	3.14E-07	193	24	Axon guidance	5.1E-05	83	18
Morphine addiction	3.14E-07	53	24	Hippo signalling pathway	8.86E-05	90	19
TGF-beta signalling pathway	1.88E-06	49	18	Biosynthesis of unsaturated fatty acids	1.69E-04	12	8
Axon guidance	1.88E-06	78	24	Adrenergic signalling in cardiomyocytes	2.52E-04	82	18
Transcriptional misregulation in cancer	2.61E-06	101	23	ErbB signalling pathway	4.00E-04	56	17
GABAergic synapse	3.09E-06	49	22	TGF-beta signalling pathway	9.09E-04	49	15
ErbB signalling pathway	3.42E-06	55	21	Fatty acid metabolism	9.29E-04	27	11
Mucin type O- Glycan biosynthesis	1.01E-05	18	13	Phosphatidylinositol signalling system	9.29E-04	51	17
FoxO signalling pathway	1.01E-05	79	21	Glutamatergic synapse	9.29E-04	66	18
Glioma	5.87E-05	40	18	Wnt signalling pathway	9.29E-04	81	19
Lysine degradation	5.94E-05	29	21	Pathways in cancer	9.29E-04	218	23
Focal adhesion	8.27E-05	118	23	Mucin type O-Glycan biosynthesis	9.53E-04	17	10
Signalling pathways regulating pluripotency of stem cells	9.09E-05	83	23	FoxO signalling pathway	1.17E-03	80	17

**Table 5:** Top 15 pathways containing the list of GO results from the Mirpath v3 webtool analysis

**TermID**	**Name**	**Frequency**	***p*-value**	**Number of genes**	**Number of miRNAs**
**Interictal vs healthy**					
GO:0048011	neurotrophin TRK receptor signalling pathway	0.07	1.22E-51	161	24
GO:0038095	Fc-epsilon receptor signalling pathway	0.14	6.77E-39	104	24
GO:0035666	TRIF-dependent toll-like receptor signalling pathway	0.04	6.03E-15	43	20
GO:0034166	toll-like receptor 10 signalling pathway	0.01	4.80E-14	38	19
GO:0,038,123	toll-like receptor TLR1:TLR2 signalling pathway	0.01	1.63E-13	39	20
GO:0034146	toll-like receptor 5 signalling pathway	0.01	1.82E-11	38	19
GO:0018279	protein N-linked glycosylation via asparagine	0.13	3.52E-07	47	19
GO:0006921	cellular component disassembly involved in execution phase of apoptosis	0.12	2.29E-06	23	19
GO:0002576	platelet degranulation	0.05	2.34E-05	32	18
GO:0050690	regulation of defense response to virus by virus	0.01	8.27E-04	16	13
GO:0061418	regulation of transcription from RNA polymerase II promoter in response to hypoxia	0.07	8.27E-04	16	16
GO:0035872	nucleotide-binding domain. leucine rich repeat containing receptor signalling pathway	0.08	1.04E-03	18	16
GO:0034199	activation of protein kinase A activity	0.02	4.86E-04	10	8
GO:0071377	cellular response to glucagon stimulus	0.06	5.90E-03	18	16
GO:0036109	alpha-linolenic acid metabolic process	0.06	6.51E-03	7	6
**Ictal vs interictal**					
GO:0048011	neurotrophin TRK receptor signalling pathway	0.07	2.39E-44	157	21
GO:0038095	Fc-epsilon receptor signalling pathway	0.14	2.95E-34	102	19
GO:0006921	cellular component disassembly involved in execution phase of apoptosis	0.12	3.28E-10	29	16
GO:0018279	protein N-linked glycosylation via asparagine	0.13	1.91E-09	54	16
GO:0038123	toll-like receptor TLR1:TLR2 signalling pathway	0.01	2.20E-09	35	15
GO:0038124	toll-like receptor TLR6:TLR2 signalling pathway	0.04	2.20E-09	35	15
GO:0034166	toll-like receptor 10 signalling pathway	0.01	4.66E-09	33	15
GO:0034146	toll-like receptor 5 signalling pathway	0.01	3.68E-07	33	15
GO:0006369	termination of RNA polymerase II transcription	0.07	1.35E-04	23	14
GO:0002576	platelet degranulation	0.05	3.17E-04	31	15
GO:0022400	regulation of rhodopsin mediated signalling pathway	0.06	4.62E-04	16	9
GO:1900740	positive regulation of protein insertion into mitochondrial membrane involved in apoptotic signalling pathway	0.02	2.12E-03	18	15
GO:0007603	phototransduction. visible light	0.07	4.18E-03	38	14
GO:0050690	regulation of defense response to virus by virus	0.01	6.52E-03	13	8
GO:0034199	activation of protein kinase A activity	0.02	7.95E-03	10	8

### Predicted targets with the highest absolute node strength values in ictal vs interictal comparison

**Fig. 4:**
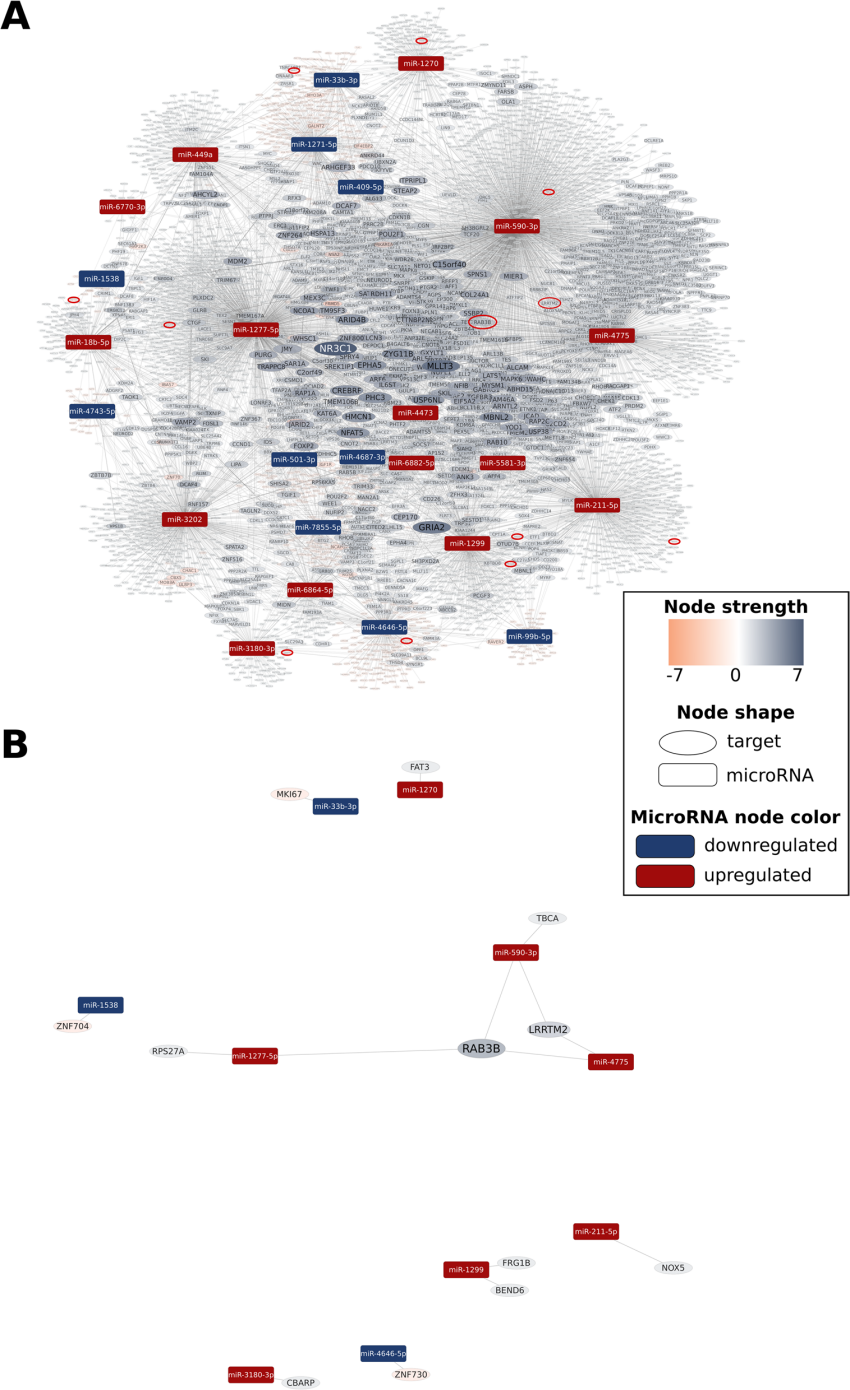
Visualisation of miRNA-mRNA interaction network (EntOptLayout) and target prediction analysis (ictal vs interictal) by miRNAtarget.com™. Rectangle and oval-shaped nodes represent miRNAs and mRNA targets of miRNAs, respectively. The node size and colour intensity of mRNA targets change according to node strength values. Down (blue)- and upregulated (red) miRNAs, suggesting a central role of downregulated (light blue) nuclear receptor subfamily 3 group C member 1 (NR3C1) and multiple upregulated (orange) mRNAs. Whole predicted miRNA-target interaction network is shown on panel **A**. To highlight important mRNA targets, targets with an absolute node strength value less than or equal to 1 (i.e. -1, 0 or 1) presented uniformly smaller and fainter. On panel **B**, a subnetwork of the whole predicted miRNA-target interaction network is shown, containing only those target mRNAs (marked with a red oval on panel **A**) and their interacting miRNAs that were validated by RNA sequencing. The same arrangement as in the whole network with a proportional magnification of the target mRNAs was applied

In ictal PBMC samples compared to the interictal subgroup, 5665 mRNAs were predicted to be regulated by the 25 significantly DE miRNAs. Among the targets with the highest absolute node strength values, NR3C1 (nuclear receptor subfamily 3 group C member 1) appears with a strength value of 7, GRIA2 (glutamate ionotropic receptor AMPA type subunit 2), and MLLT3 (MLLT3 super elongation complex subunit) with a strength value of 6. 32 upregulated targets with a node strength of -2, like FN1 (Fibronectin 1) and CBX5 (Chromobox 5) were detected in ictal-interictal comparison (Table 6; the intersect of predicted targets and DE mRNAs in S5). DE miRNAs with their predicted interactions are shown in Fig. 4.

**Table 6:** List of predicted targets up- or downregulated by DE miRNAs in interictal vs healthy and ictal vs interictal comparison, with the highest absolute node strength values. The intersect of predicted targets and DE mRNAs is available in Table S4-6

**Interictal vs Healthy**			**Ictal vs Interictal**		
**Official Symbol**	**Official Full Name**	**Node strength**	**Official Symbol**	**Official Full Name**	**Node Strength**
**Targets predicted to be downregulated**					
CADM2	cell adhesion molecule 2	4	NR3C1	nuclear receptor subfamily 3 group C member 1	7
PLEKHM3	pleckstrin homology domain containing M3	4	GRIA2	glutamate ionotropic receptor AMPA type subunit 2	6
MEF2C	myocyte enhancer factor 2C	4	MLLT3	MLLT3 super elongation complex subunit	6
BBX	BBX high mobility group box domain containing	4			
RIMKLB	ribosomal modification protein rimK like family member B	4			
HACE1	HECT domain and ankyrin repeat containing E3 ubiquitin protein ligase 1	4			
**Targets predicted to be upregulated**					
CCNT2	cyclin T2	-6	32 targets with a node strength of -2		
KLHL15	kelch like family member 15	-8

In addition, the intersect of 6781 predicted targets 31 DE miRNAs data in ictal vs healthy comparison and DE mRNAs are enlisted in Table S6. The visualisation of DE miRNAs with their predicted interactions in ictal vs. healthy interaction can be found in Figure S2.

### Gene Ontology enrichment analysis of predicted mRNAs

To explore biological processes modified by migraine in headache-free (interictal) period and during headache (ictal), GO enrichment analyses of all predicted mRNAs were performed. The result of the GO enrichment analysis showed that DE miRNA-targeted mRNAs were significantly associated with several metabolic regulation processes and negative regulation of platelet activation when the interictal group was compared to healthy samples. Besides, regulation of nuclear-transcribed mRNA catabolic process, neuron development, and neuron differentiation seem to be affected by migraine (Table 7).

**Table 7:** Gene Ontology (GO) enrichment analysis (biological processes) of all miRNA targets in interictal vs healthy and ictal vs interictal comparisons. The top ten predicted up-and downregulated processes with the highest fold enrichment values are presented here, where sorting is based on the fold enrichment of the most specific subclasses

**Interictal vs Healthy**			**Ictal vs Interictal**		
**GO biological process**	**Fold Enrichment**	**FDR**	**GO biological process**	**Fold Enrichment**	**FDR**
**Targets predicted to be downregulated**					
flavonoid glucuronidation (GO:0052696)	10.28	7.09E-04	postsynaptic specialization organization (GO:0099084)	3.47	3.09E-02
negative regulation of cellular glucuronidation (GO:2001030)	10.28	2.04E-03	regulation of nuclear-transcribed mRNA catabolic process, deadenylation-dependent decay (GO:1900151)	3.47	3.09E-02
negative regulation of glucuronosyltransferase activity (GO:1904224)	10.28	2.03E-03	postsynaptic density organization (GO:0097106)	3.47	3.08E-02
regulation of glucuronosyltransferase activity (GO:1904223)	10.28	2.02E-03	negative regulation of smooth muscle cell migration (GO:0014912)	3.30	1.49E-02
regulation of cellular glucuronidation (GO:2001029)	9.14	3.21E-03	hippo signalling (GO:0035329)	2.85	3.17E-02
xenobiotic glucuronidation (GO:0052697)	8.41	1.88E-03	regulation of mesenchymal cell proliferation (GO:0010464)	2.72	1.91E-02
flavonoid metabolic process (GO:0,009,812)	6.43	3.10E-03	roof of mouth development (GO:0060021)	2.47	7.20E-05
cellular glucuronidation (GO:0052695)	6.28	1.67E-03	regulation of smooth muscle cell migration (GO:0014910)	2.41	4.74E-03
uronic acid metabolic process (GO:0006063)	5.36	2.17E-03	regulation of dendritic spine morphogenesis (GO:0061001)	2.41	1.85E-02
glucuronate metabolic process (GO:0019585)	5.36	2.16E-03	positive regulation of epithelial to mesenchymal transition (GO:0010718)	2.36	2.89E-02
**Targets predicted to be upregulated**					
negative regulation of platelet activation (GO:0010544)	3.35	3.79E-02	regulation of cell projection organization (GO:0031344)	2.03	3.79E-02
G1 phase (GO:0051318)	3.28	3.00E-02	neuron development (GO:0048666)	1.95	2.14E-02
mitotic G1 phase (GO:0000080)	3.28	3.00E-02	intracellular protein transport (GO:0006886)	1.79	3.90E-02
regulation of transcription involved in G1/S transition of mitotic cell cycle (GO:0000083)	3.27	1.87E-03	neuron differentiation (GO:0030182)	1.78	3.58E-02
response to muscle stretch (GO:0035994)	3.22	2.35E-02	generation of neurons (GO:0048699)	1.78	1.80E-02
negative regulation of cyclin-dependent protein kinase activity (GO:1904030)	3.18	6.66E-03	neurogenesis (GO:0022008)	1.72	1.79E-02
regulation of sister chromatid cohesion (GO:0007063)	3.13	3.85E-02	intracellular transport (GO:0046907)	1.62	3.97E-02
negative regulation of cyclin-dependent protein serine/threonine kinase activity (GO:0045736)	3.11	1.12E-02	cellular macromolecule localization (GO:0070727)	1.61	3.15E-02
regulation of histone H3-K9 methylation (GO:0051570)	3.09	3.02E-02	cellular protein localization (GO:0034613)	1.60	3.66E-02
positive regulation of pri-miRNA transcription by RNA polymerase II (GO:1902895)	3.05	2.47E-03	nitrogen compound transport (GO:0071705)	1.56	3.40E-02

### Validation of predicted mRNA targets miRNAs based on mRNA sequencing data

In interictal PBMC samples compared to healthy samples, out of 7012 predicted targets, 54 can also be found in DE mRNA data. In this comparison, 31 targets showed expression changes in the predicted direction, like TNF (tumor necrosis factor), SOCS3 (suppressor of cytokine signalling 3), numerous chemokines such as IL6 (interleukin-6), and SOD2 (superoxide dismutase 2), with a role in oxidative phosphorylation (Table 8). Of 5665 predicted targets, 26 can also be found in DE mRNA data in the ictal-interictal analysis. In this settlement, 12 targets showed expression changes in the predicted direction, e.g., RAB3B. (Ras-related protein Rab-3B), LRRTM2 (Leucine-Rich Repeat Transmembrane Neuronal 2) (Table 9).

**Table 8:** Common targets, with similar changes in mRNA- and small RNA sequencing data for validation of predictions at mRNA level in interictal vs healthy comparison. Description and pain phenotype matches based on Human Pain Gene [53], Pain Research Forum [54], DisGeNET [55], and GeneCards [56], complemented with other literature data. logFC: logarithm of the fold change of measured mRNA data

Interictal vs Healthy				
**Target**	**logFC**	**Node Strength**	**Description**	**Pain/inflammation related disease/processes** **(if applicable)**
PLCXD2	-1.337	1	phosphatidylinositol specific phospholipase C X domain containing 2	
TNF	2.814	-1	tumor necrosis factor	migraine, high pain and high fatigue, cancer pain [53], painful neuropathy [54], rheumatoid arthritis [55], Crohn disease [55]
EGR1	2.616	-1	early growth response 1	regulates proteins involved in inflammation [56]
EREG	3.063	-1	epiregulin	temporomandibular disorder [53]
CD83	2.504	-1	CD83 molecule	upregulated by oxidative stress; maturation marker, antiinflammatory effects [57, 58]
NFKBIA	1.861	-1	NFKB inhibitor alpha	cancer pain [53]
IER3	1.810	-1	immediate early response 3	sarcoidosis [55]
TNFAIP6	2.464	-1	tumor necrosis factor alpha-induced protein 6	rheumatoid arthritis [59]
ID1	1.723	-1	inhibitor of DNA binding 1, HLH protein	response to oxidative stress [60]
OSR2	1.936	-1	odd-skipped related transciption factor 2	upregulated in mirror image pain in rat CRPS model [61]
NR4A2	1.752	-1	nuclear receptor subfamily 4 group A member 2	associated with dopaminergic neuron differentiation and dopamine biosynthetic processes [62], neuroinflammation [63] mediates production of inflammatory cytokines[64]
CNTNAP3	2.178	-1	contactin associated protein-like 3	Crohn’s Disease [65],
FOSB	1.524	-1	FosB proto-oncogene, AP-1 transcription factor subunit	chronic pain [66], inflammatory pain [67]
IL6	1.826	-1	interleukin 6	neuraxial pain, analgesia, musculoskeletal pain, cancer pain, arthritis; irritable bowel syndrome; sciatica intervertebral disc disease pain [53]
EGR3	1.694	-1	early growth response 3	neuropathy[56]
DUSP1	1.265	-1	dual specificity phosphatase 1	antiinflammatory in neuropathic pain [68], alleviates neuroinflammation and neuronal injury [69]
SOCS3	1.284	-1	suppressor of cytokine signalling 3	regulates cytokine signal transduction[56], chronic pain [66]
SAT1	1.147	-1	spermidine/spermine N1-acetyltransferase 1	neuroinflammation [70]
RGS1	1.681	-1	regulator of G protein signalling 1	undifferentiated spondylarthritis [71]
PLAU	1.312	-1	plasminogen activator, urokinase	psoriasis, ulcerative colitis, Crohn's disease, inflammatory bowel disease [55]
MIPOL1	1.252	-1	mirror-image polydactyly 1	nasopharyngeal carcinoma [72]
JUNB	1.059	-1	JunB proto-oncogene, AP-1 transcription factor subunit	psoriasis [73]
SGK1	0.975	-1	serum/glucocorticoid regulated kinase 1	pain developement [74]
MAP3K7CL	0.878	-1	MAP3K7 C-terminal like	non‐small cell lung cancer [75]
CXCL8 (IL8)	2.806	-2	C-X-C motif chemokine ligand 8 (interleukin 8)	cancer pain [53]
PLAUR	1.428	-2	plasminogen activator, urokinase receptor	inflammatory bowel disease [76]
DUSP2	1.521	-2	dual specificity phosphatase 2	endometriosis, cancer, immune and inflammatory resonses [77]
RBKS	1.202	-2	ribokinase	
GXYLT2	1.888	-3	glucoside xylosyltransferase 2	ulcerative colitis [78]
SOD2	1.429	-4	superoxide dismutase 2	migraine [55]
SOCS1	1.033	-4	suppressor of cytokine signalling 1	Crohn's disease, psoriasis [55]

**Table 9:** Common targets, with similar changes in mRNA- and small RNA sequencing data for validation of predictions at mRNA level in ictal vs interictal comparison. Description and pain phenotype matches based on Human Pain Gene [53], Pain Research Forum [54], DisGeNET [55], and GeneCards [56], complemented with other literature data. logFC: logarithm of the fold change of measured mRNA data

Ictal vs Interictal				
**Target**	**logFC**	**Node** **strength**	**Description**	**Pain/inflammation related disease/processes** **(if applicable)**
RAB3B	-1.370	3	RAB3B, member RAS oncogene family	psoriasis [56], multiple sclerosis [79]
LRRTM2	-1.051	2	leucine rich repeat transmembrane neuronal 2	excitatory synaptic transmission [80], AMPA receptor transmission [81],
NOX5	-1.051	1	NADPH oxidase 5	oxidative stress [82]
FAT3	-1.003	1	FAT atypical cadherin 3	neuropathy [55]
CBARP	-0.955	1	CACN subunit beta associated regulatory protein	Negatively regulates voltage-gated calcium channels [56]
BEND6	-0.890	1	BEN domain containing 6	epilepsy [66], inhibits Notch signalling in neural stem cells, thereby opposing their self-renewal and promoting neurogenesis [56]
RPS27A	-0.675	1	ribosomal protein S27a	microglia activation in neurodegenrative diseases [83], upregulated in enhanced pain sensitivity [62]
TBCA	-0.639	1	tubulin folding cofactor A	brain injury, ischemia [66]
FRG1B	-0.620	1	FSHD region gene 1 family member B, pseudogene	
ZNF730	1.175	-1	zinc finger protein 730	transcriptional regulation [56]
ZNF704	0.967	-1	zinc finger protein 704	transcription factor [56], potential candidate gene for aging in women [84]
MKI67	0.826	-1	marker of proliferation Ki-67	cellular proliferation [56]

## Discussion

The present results obtained from a self-controlled study design provide the first evidence for disease and headache-specific miRNA patterns in PBMC of migraineurs. Our findings can help to identify key elements of the pathophysiological pathways, determine potential novel diagnostic and prognostic biomarkers and drug targets. The discovery of the small non-coding RNAs [25], together with the use of PBMCs, has offered new opportunities in this area [10].

In the interictal PBMC samples, 31 miRNAs were DE compared to healthy controls, including hsa-miR-5189-3p, hsa-miR-96-5p, hsa-miR-3613-5p, hsa-miR-99a-3p, and hsa-miR-542-3p. hsa-miR-5189-3p closely correlates with spinal cord injury and is part of a set of transcripts proposed to have a role in classifying the disease [85]. The miR-96 and its miR-183 family have been observed to be regulated in diverse regions of the somatosensory nociceptive pathway in different chronic pain conditions in rodents [86]. The miR-183 cluster is distinctly enriched in sensory organs. It may influence the neuronal changes associated with the development and maintenance of chronic pain conditions by coordinately regulating multiple diverse nociceptive genes [87]. Circulating hsa-miR-3613-5p increased in severe axial pain after accident [88] and decreased in endometriosis [89, 90]. Serum hsa-miR-542-3p was found to be a part of miRNA signatures for endometriosis diagnosis [91]. The hsa-miR-99a-3p was downregulated in salivary exosomal miRNAs of burning mouth syndrome patients [92].

Our results revealed GABAergic synaptic changes in both interictal and ictal samples of migraineurs compared to healthy controls. The abnormalities of GABAergic signalling in migraine pathophysiology have been widely investigated in the literature [93]. Serum miRNA results also have suggested that targeting the GABA system might have therapeutical relevance [27]. Interestingly, but not surprisingly, the rhodopsin-mediated signalling pathway and phototransduction were involved as attack specific alterations (ictal-interictal comparison). Photophobia is a light-induced phenomenon linked with migraine and intensifying headache pain [94]. Although migraine research primarily focuses on the affected melanopsin-related pathways [95], it is clear that abnormal light processing is present in these patients. However, the reflection of these alterations in peripheral blood samples could be an exciting question in further research.

Similar to our PBMC results, differentially expressed miR-155 and let-7 g levels related to endothelial functions were described in migraineurs’ serum samples during ictal and interictal periods [96]. Another member of the let-7 family, let-7b, was found to be downregulated in migraine patients’ PBMC samples [28].

The top DE miRNAs during headache attacks compared to the self-control interictal samples were the following: hsa-miR-3202, hsa-miR-7855-5p, hsa-miR-6770-3p, hsa-miR-1538, hsa-miR-409-5p. Among exosomal miRNAs, hsa-miR-3202 was dysregulated in mild traumatic brain disorders [97], and hsa-miR-409-5p was DE in complex regional pain syndrome [98]. The latest was downregulated after contusion spinal cord injury, while its overexpression could promote recovery [91]. hsa-miR-7855-5p is known to be hypoxia-responsive [99], suggesting a relation to oxidative stress, and hsa-miR-1538 modifies cell response to oxidative stress [100]. hsa-miR-6770-3p decreased in chronic periodontitis as a potential biomarker [92]

In both comparisons, the list of top predicted targets with the highest absolute node strength value consists of a few mRNAs linked to the pathophysiological mechanisms of migraine. GRIA2 and NR3C1, involved in glutamate and glucocorticoid signalling, respectively, appear to be specific to the headache phase in our data set. Our results are supported by the demonstration of glutamatergic neurotransmission changes in trigeminovascular activation and central sensitisation [101, 102]. However, a case–control study did not find an association between migraine and GRIA2 polymorphisms [103]. DNA methylation of the NR3C1 gene is the focus of epigenetic literature [104, 105]. GWAS studies link the transcription factor MEF2, which was downregulated in our study, to neuroinflammatory processes [106], epilepsy [107], psychological and metabolic features [108, 109].

Pathway analysis pointed out TLR (toll-like receptor) signalling pathways involved in both the headache-free state and headache. TLRs are transmembrane receptors playing a crucial role in innate immune response and inflammation, which activate the NF-κB and interferon regulatory factors resulting in proinflammatory cytokine production like TNFα, IL1, IL6, and IL12. Altered interleukin and TNFα levels in migraine have been demonstrated in the literature [110–112]. Our miRNA target prediction results are in line with these data. IL-6 and TNFα were validated by comparing to mRNA levels demonstrated in our previous paper [11]. Although the involvement of TLR4 signalling in initiating and maintaining migraine-like behaviour in mice and inducing hyperalgesia in rats has been proposed [113–117], this is the first study that links the TLR pathway specifically to migraineurs. In addition, there is evidence suggesting a strong TLR-4/miRNA interplay, which might be a possible target for modern immunotherapy [118].

Among validated targets describing the migraine disease in the attack-free period, EGR1 (early growth response gene 1) was enlisted, which can regulate multiple aspects of synaptic plasticity [119] [120]. Several validated targets line up as regulators and participants of the immune system and inflammatory pathways, like NFKBIA (NFKB inhibitor alpha) and IER3 (early response 3) [121, 122]. TNFAIP6 (TNF Alpha Induced Protein 6) might be a potential miRNA target with anti-inflammatory properties [59]. NR4A2 (Nuclear Receptor Subfamily 4 Group A Member 2), through the regulation of various signals, inhibits the expression of proinflammatory mediators and plays a neuroprotective role [63]. EGR3 (Early Growth Response 3) is essential for controlling inflammation and antigen-induced immune cell proliferation [123]; it suppresses SOCS1, SOCS3 (cytokine signalling-1 and 3), regulating cytokine or hormone signalling. The enzyme encoded by the SAT1 (Spermidine/Spermine N1-Acetyltransferase 1) gene catalyses the acetylation of spermidine and spermine, thus mediating polyamine metabolism. This data underlines our previous findings [11], where we described alterations in spermin and spermidine metabolites in migraineurs plasma samples. The role of oxidative stress and mitochondrial dysfunction in migraine susceptibility and headache generation has also been demonstrated in our recent work [11]. Sgk (serum- and glucocorticoid-inducible protein kinase) [124] and SOD (superoxide dismutase) [125] increased in the interictal samples when compared to controls, underlining the importance of these pathways. In addition, the ictal vs interictal comparison enlisted downregulation of the transmembrane signalling enzyme NOX5 (NADPH oxidase 5) [119].

## Conclusions

We provide here the first miRNA profile of migraineurs in headache-free periods and during attacks compared to healthy controls suggesting potential disease- and pain-specific pathophysiological mechanisms. Predicted mRNA targets of differentially expressed miRNAs confirmed and validated by the transcriptomics analysis reveal the involvement of inflammatory and immune mechanisms, cytokine and chemokine signalling, and oxidative stress.

## Supplementary Information

**Additionalfile 1: Supplementary Table 1.** Differentially expressed (DE)miRNA list of PBMC small RNA-seq data of the interictal vs healthy comparison. **Supplementary Table 2.** Differentially expressed (DE) miRNA list of PBMC small RNA-seq data of the ictal vs interictal comparison. **Supplementary Table 3.** Differentially expressed (DE) miRNA list ofPBMC small RNA-seq data of the ictal vs healthy comparison. **Supplementary Table 4.** List of intersectof predicted targets up- or downregulated by DE miRNAs and DE mRNAs ininterictal vs healthy comparison. **Supplementary Table 5.** List of intersect of predicted targets up- ordownregulated by DE miRNAs and DE mRNAs in ictal vs interictal comparison. **Supplementary Table 6. **List of intersect of predicted targets up- ordownregulated by DE miRNAs and DE mRNAs in ictal vs healthy comparison. **Supplementary Figure 1.** Heat map visualization of DE miRNAs in ictal vs. healthy comparison. **Supplementary Figure 2.** Visualization of DE miRNAs with predicted interactions in ictal vs. healthy interaction. 

## Data Availability

All RNA-Seq data sets generated as part of this study will be publicly available at the European Nucleotide Archive (https://www.ebi.ac.uk/ena), under accession number PRJEB46142.

## Abbreviations

ANOVA: Analysis of variance
BMI body: Mass index
CGRP: Calcitonin gene-related peptide
DE: Differentially expressed
EDTA: Ethylenediaminetetraacetic acid
FC: Fold change
FDR: False discovery rate
GO: Gene Ontology
GWAS: Genome-wide association studies
KEGG: Kyoto Encyclopedia of Genes and Genomes
PACAP: Pituitary adenylate cyclase-activating polypeptide
PBMC: Peripheral blood mononuclear cells
TNF: Tumor necrosis factor
VAS: Visual analogue scale
